# Prostate Cancer in Sexual Minorities: Epidemiology, Screening and Diagnosis, Treatment, and Quality of Life

**DOI:** 10.3390/cancers15092654

**Published:** 2023-05-08

**Authors:** Omid Yazdanpanah, David J. Benjamin, Arash Rezazadeh Kalebasty

**Affiliations:** 1 Division of Hematology and Oncology, Department of Medicine, UC Irvine Medical Center, Orange, CA 92868, USA; arez@hs.uci.edu; 2 Hoag Family Cancer Institute, Newport Beach, CA 92663, USA; david.benjamin@hoag.org

**Keywords:** prostate cancer, sexual minorities, disparities, quality of life

## Abstract

**Simple Summary:**

Prostate cancer is the most common cancer among men. There is growing recognition of disparities faced in diagnosis, treatment and post-treatment outcomes in sexual minorities, including gay and bisexual men, as well as transgender women. Although it is unclear whether sexual minorities have a higher incidence of prostate cancer compared to heterosexual men, several qualitative and quantitative studies have established worse quality-of-life outcomes for sexual minorities following prostate cancer treatment. Further studies are urgently warranted in this growing population in order to provide the best care to sexual minorities with prostate cancer.

**Abstract:**

Prostate cancer has the highest incidence among all cancers in men. Sexual minorities, including gay and bisexual men, as well as transgender, were previously a “hidden population” that experienced prostate cancer. Although there continues to remain a paucity of data in this population, analyses from studies do not reveal whether this population is more likely to endure prostate cancer. Nonetheless, several qualitative and quantitative studies have established worse quality-of-life outcomes for sexual minorities following prostate cancer treatment. Increased awareness of this previously “hidden population” among healthcare workers, as well as more research, is warranted to gain further understanding on potential disparities faced by this growing population.

## 1. Introduction

Prostate cancer is the most common cancer among men, and in 2022 alone, was responsible for 11% of cancer-related deaths in the United States [1]. Sexual minorities refer to individuals who identify as lesbian, gay, bisexual, transgender/transsexual, queer/questioning, or any other non-heterosexual identity [2]. Despite the high prevalence of prostate cancer, its impact on sexual minority patients, including gay and bisexual men (GBM), or transgender women (TW), is not clearly understood. As such, improved understanding surrounding the screening, diagnosis, and treatment patterns of prostate cancer in this patient population is urgently warranted.

Members of sexual minority communities have historically experienced unique, unaddressed healthcare needs and obstacles [3]. Screening, diagnosis, and treatment of prostate cancer can present additional challenges for sexual minority patients, including the potential for discrimination and stigma in healthcare settings [4]. The use of prostate specific antigen (PSA) screening may also present unique challenges for sexual minority patients, as some may not have regular access to care, may be hesitant to disclose their sexual identity to healthcare providers, or reveal previous hormonal treatments [5,6]. The impact of prostate cancer on quality of life is also a significant concern for sexual minority patients, as the disease and its treatment can have a profound effect on sexual function and intimacy, which may be particularly important for this population [7].

The objective of this review is to provide an overview of prostate cancer burden in men who identify as gay or bisexual, as well as in transgender women. We summarize the existing evidence for the development and incidence of prostate cancer, screening, diagnostics and treatment options in sexual minorities. Additionally, we discuss the resultant disparities on quality-of-life outcomes in this population.

## 2. Sexual Minorities and Access to Health Care

The lesbian, gay, bisexual, transgender/transsexual, and queer/questioning (LGBTQ) community, is a growing, yet medically-underserved, population [8]. In addition to certain type of diseases, such as sexually transmitted diseases (STDs), rejection, anxiety, and depression, sexual minorities also experience significant health disparities when compared to heterosexual people [9]. Studies have shown that patients who identify as non-heterosexual report lower levels of access to health services such as health insurance, having a primary health care provider, and receiving culturally appropriate services [3].

Sexual minorities are less likely to have health insurance coverage despite significant gains in coverage and having a usual source of care through the implementation of the Affordable Care Act (ACA) [10]. Dahlhamer and colleagues investigated the association between sexual orientation with barriers to health care, and found that the likelihood of gay adults delaying or not receiving care due to cost and non-cost reasons was more than twice as high as heterosexual adults. Gay men also had more difficulty finding a healthcare provider (8.5% vs. 4.2%) than their heterosexual counterparts [11].

There are also barriers in gaining culturally appropriate health services including an insufficient number of culturally competent providers educated in LGBTQ needs [12]. Fear of stigmatization may make LGBTQ patients more likely to remain silent about pertinent health issues. As such, these structural obstacles contribute to the avoidance or delay of seeking care in spite of the ongoing need for health care in the LGBTQ community [13].

Discrimination based on sexual orientation and gender identity is a concern among this patient population. A systematic review by Ayhan et al. indicated that the rate of discrimination experienced by sexual and gender minority (SGM) patients has been reported between 2% to 42% in different studies [14]. In another study by Casey et al., 16% of SGM adult participants reported experiencing regular discrimination [15]. The negative experiences of these patients during health care encounter can lead to changing physicians, gaps in healthcare, and in some cases, a loss of trust in the health care system.

There has recently been an increased focus in better understating these obstacles and deficiencies in access to health care for sexual minority groups and attempts to overcome these barriers. In an effort to enhance comprehension of the sexual minority health needs, the US Office of Disease Prevention and Health Promotion has included the exploration of LGBT health in their Healthy People 2020 initiative as a public health objective [16]. More research is required in this domain to elucidate the inequities in the healthcare system for SGM populations, with the aim of influencing policy reform.

## 3. Prostate Cancer Epidemiology in Sexual Minorities

Prostate cancer is the most commonly diagnosed malignancy in men and is the second-leading cause of cancer-related death following lung cancer [1]. It is difficult to assess the exact incidence and prevalence of prostate cancer in sexual and gender minority groups, including GBM and TW, for multiple reasons. A substantial proportion of these individuals might be reluctant to disclose their sexual identity. In addition, large scale cancer surveillance surveys do not routinely collect sexual orientation and practices [17].

Prostate cancer is mostly diagnosed in the 6th and 7th decade of life. Several studies suggest that the mean age of prostate cancer diagnosis is younger in gay men than in heterosexuals, although it still mainly affects middle-aged populations in their 60s [18]. However, several factors need to be considered which might have led to sampling bias in studies of gay men with prostate cancer. A significant, but unknown, number of sexual minorities died during the AIDS epidemic of the 1980s. Moreover, individuals who have gone on to be diagnosed with disease could have limitations reporting their sexual orientation and related health concerns due to long-standing barriers in a historically heteronormative system [4]. Studies have shown that older men from sexual minority groups might be less likely to declare their sexual orientation as gay, which can also cause bias in sampling. Collected data from Gallup surveys from more than 1.6 million adults between 2012 and 2016 revealed that while 2.4% for baby boomers (born between 1946 and 1964) identified as LGBT, this number was 7.8% for millennials (born between 1980 and 1998) [19].

It is estimated that sexual minority groups compose 1.5–6.0% of the US population. Therefore, approximately 49,500–198,000 gay and bisexual men, and transgender women are living with prostate cancer in the USA [20,21]. As sexual and gender minority groups gain greater acknowledgement, a new area of research has emerged to investigate the epidemiology of cancer within this demographic [22]. In a study of 51,233 individuals by the California Health Interview Survey in 2011, Boehmer et al. reported a significantly lower reported prostate cancer in gay men (5.3%) compared with heterosexual men (16.5%) and bisexual men (14.3%) [17]. Given that GBM are at a higher risk for HIV (human immunodeficiency virus) and other sexually transmitted infections (STIs), any comparative research on prostate cancer incidence across different sexual orientations should account for the participants’ HIV and STI history [23].

Several case-control studies have attempted to investigate if GBM are disproportionately at greater risk for prostate cancer with differing conclusions among these studies [24]. Mandel et al. included 250 cases of prostate cancer, 238 hospital controls, and 240 neighborhood controls in their project. They found that prostate cancer group members were more likely to have a history of STIs and same-sex partners than the control group [25]. Research by Rosenblatt et al. with 753 cases of prostate cancer and 703 control cases revealed that lifetime numbers of female partners and history of gonorrhea increased the risk of prostate cancer. However, there was no association between sexual orientation, anal sex, or a history of male partners with prostate cancer [26]. According to the case-control study performed by Spence et al. in Canada with more than 3000 participants, a history of STIs or identifying as GBM did not significantly increase the risk of prostate cancer, but men who had 20 or more lifetime male sexual partners had a slightly higher risk [27]. Further research is warranted to determine which, if any, factors may contribute to disparities in incidence of prostate cancer in sexual minorities.

Santillo and Lowe reviewed several risk factors for prostate cancer development among gay men. They suggested that theoretically exogenous use of testosterone and anabolic steroids, use of finasteride for hair loss, and high-fat diet may potentially increase the risk of prostate cancer in this community. Anal sex and its impact on prostate specific antigen (PSA) testing, HIV status and antiretroviral (ARV) treatment, and patient-doctor communication are additional factors that may impact epidemiology of prostate cancer in gay males [28].

The association between HIV/AIDS (acquired immunodeficiency syndrome) and prostate cancer have previously been investigated. Several studies have proposed that HIV-positive men are at higher risk of prostate cancer than HIV-negative [29,30]. However, studies performed during the era of ARV treatment and PSA screening have shown the opposite trend. The US HIV/AIDS Cancer Match Study, which studied men who met the clinical definition for AIDS, found no difference in prostate cancer incidence compared to the general population before the introduction of the PSA test and ARV treatment (before 1992). However, during the PSA era (1992–2007), there was a significant reduction in the risk, with a twofold decrease among men with AIDS. Of note, this study also revealed that PSA testing rates were lower among low-income HIV-infected men [31].

**Figure 1:**
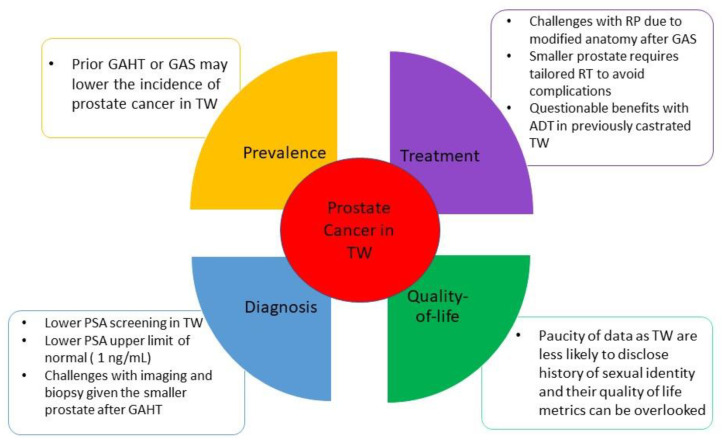
Specific considerations for prostate cancer in transgender women. Abbreviations: ADT: androgen deprivation therapy. GAHT: gender-affirming hormonal therapy. GAS: gender-affirming surgery. PSA: prostate specific antigen. RP: radical prostatectomy. RT: radiation therapy. TW: transgender women.

Incidence of prostate cancer in the transgender women community (i.e., who are assigned male at birth) is poorly understood. Bertoncelli Tanaka et al. performed a non-systematic review of the literature related to PC in transgender women by including 10 case reports, four specialist opinion papers, six cohort studies, and four systematic reviews. They concluded that the likelihood of developing prostate cancer for TW who are not receiving gender-affirming hormone therapy (GAHT) or who have not undergone gender-affirming surgery (GAS), and gender non-conforming individuals (who may never commence GAHT or have GAS) is similar to that of cis-gender males. However, transgender women on GAHT or following GAS have lower incidence of prostate cancer than age-matched cis-male counterparts [5]. Given the paucity of data in the literature regarding incidence of prostate cancer in transgender women, it is challenging to draw conclusions regarding potential disparities in incidence of prostate cancer in this population (Figure 1).

## 4. Prostate Cancer Screening and Diagnosis in Sexual Minorities

Prostate cancer screening using PSA testing is intended to detect the early-stage cancer that may be treated and potentially cured. Nonetheless, controversy surrounding PSA screening remains, regarding whether testing reduces disease-specific morbidity and/or mortality in the general population, or merely leads to invasive diagnostic procedures and associated complications of treatments without prolonging life [32]. In 2012, the US Preventive Services Task Force (USPSTF) allocated a grade D recommendation to PSA testing (recommendation against PSA-based screening for prostate cancer) which was later changed to grade C (advocating for an individualized approach to screening) in 2018 for men aged 55–69 years. Both USPSTF and the American Urological Association (AUA) emphasize the necessity of a risks versus benefits discussion and shared decision-making with patients [33,34]. However, the pattern of prostate cancer screening in sexual minorities remains obscure.

Ma et al. carried out a cross-sectional study to evaluate self-reported PSA screening and decision-making among LGBT groups in the US. The study had a weighted estimate of more than a million individuals who identified as gender or sexual minorities. Their study revealed that select gay and bisexual individuals are more inclined to participate in PSA screening recommended by their clinicians [35]. This finding was in line with an earlier report indicating that men who do not identify as heterosexual may undergo more rigorous screening procedures. Although the exact reason for higher participation of select gay populations in prostate cancer screening is not clear, higher concern about loss of ejaculation after prostatectomy was suggested [36]. Berg et al. found in a separate study that gay men are more likely to be screened for prostate cancer and discuss the advantages of PSA testing with the physician prior to the test. It was also shown that gay men who were screened for prostate cancer were younger than their heterosexual counterparts with a median age of 58 years (52–66) versus 64 years (56–71) [6].

PSA screening literature among gay and bisexual men lacks consistent collection and analysis of mediators and confounding variables, which may impact the results. In the California Health Interview Survey, gay/bisexual men had a lower likelihood of having up-to-date PSA testing compared with heterosexuals, when adjusted for race/ethnicity, education, or language proficiency. Use of this test by gay/bisexual African Americans was 12–14% less than that of straight African Americans and 15–28% less than that of gay/bisexual Whites in this study [37]. In another study by Fredriksen-Goldsen et al., initial analyses suggested that sexual minority men had a significantly lower odds of having PSA testing compared to heterosexual men. However, after adjusting for sociodemographic variables, the observed difference was no longer statistically significant [8]. Further epidemiological studies are needed to fully comprehend the relationships between sexual orientation, demographic characteristics, and PSA screening.

Transgender participants in Ma et al.’s study were less likely to have PSA screening [35]. This is supported by findings from two additional studies that indicated that transgender women were less likely than cis-gendered men to ever have a risks versus benefits discussion about PSA screening with healthcare providers [38,39]. It is plausible that transgender women may find it challenging to disclose their personal sexual identity history to primary care physicians, leading to the possibility of not being offered the opportunity to talk about PSA screening when it is appropriate [5]. There is also a lack of international consensus in different guidelines regarding screening of TW for prostate cancer. Experts have recommended PSA testing discussion before starting GAHT or during GAHT should be offered to those who are eligible based on national guidelines similar to a cis-gendered man [40].

Nonetheless, given that PSA levels drop significantly after initiating GAHT, the upper limit of normal for PSA in the TW population is considered 1 ng/mL [5,40]. Prostate biopsy in TW following GAS is not contraindicated as studies indicate biopsy can safely be done with a transneovaginal ultrasound probe similar to standard transrectal ultrasound and biopsy. However, anatomical modifications following GAS and also the smaller prostate size secondary to GAHT needs to be taken into consideration [41]. Cases with PSA more than 1 ng/mL can be evaluated with multiparametric prostate MRI (MP-MRI) and the ones with PI-RADS (Prostate Imaging Reporting and Data System) ≥3 lesion will be considered for biopsy [42]. Hugosson et al. found in a study published in NEJM that MRI-directed targeted biopsy is helpful to detect prostate cancer in a low PSA population; although it can miss clinically non-significant diseases which can reduce the risk of overdiagnosis [43]. PSA density (PSAd) can serve as a useful tool in addition to PI-RADS for identifying individuals who require a biopsy to diagnose prostate cancer. Friesbie et al. demonstrated that PSAd, with a cutoff of 0.1 ng/mL/cc, can stratify the risk of prostate cancer in a complementary way with prostate MP-MRI [44].

## 5. Prostate Cancer Treatment in Sexual Minorities

Treatment options for localized prostate cancer include external beam radiation therapy (RT) with or without brachytherapy, brachytherapy alone, radical prostatectomy, or active surveillance. These options are recommended based on the risk stratifications provided by the American Society of Clinical Oncology, American Urological Association, American Society for Radiation Oncology, and Society of Urologic Oncology [45,46]. The decision regarding treatment is arrived at after a discussion with the patient, weighing the potential risks against the benefits. A study utilizing an online prostate cancer discussion board demonstrated that gay men were more worried about the negative impacts of treatment and the availability of psychological and emotional support, whereas straight men were more interested in exploring the different treatment options available to them [47]. These concerns by gay men may impact treatment preferences, but additional research is required to substantiate this possibility.

It is unclear if treatment patterns in the GBM community differ from heterosexual men. For example, there have been reports that Gleason scores were found to be significantly lower in GBM treated for prostate cancer than in heterosexual men [24,36]. In a study performed by Murphy et al. in Chicago, it was found that men who are HIV-positive are equally likely to receive treatment for prostate cancer. However, they are less likely to undergo a radical prostatectomy and more likely to receive overtreatment compared to men who are HIV-negative [48]. Use of ARV treatment for HIV has been suggested to be protective in prostate cancer [49].

Wassersug et al. included 460 heterosexual and 92 non-heterosexual men in their study and they observed no difference in treatment pattern between heterosexual and non-heterosexual [36]. In a study by Hart et al., it was found that the rates of prostatectomy, external beam radiation, and ADT in gay men were 55.4%, 27.2%, and 25%, respectively. While there was no control group of heterosexual men included in the study, the rates of treatment choices in gay men were similar to those observed in the general population [18]. Another cross-sectional study with a cohort of gay men, which also lacked a heterosexual control group, found that these men had slightly higher rates of surgical treatment (60.4%) and similar rates of radiotherapy (27%) compared to the general population [50]. Ussher et al. found in their study that gay and bisexual men were slightly less likely to receive radiotherapy than heterosexual men [51]. Although these studies provide some understanding of the patterns in treatments, they do not explain the decision-making process involved in treatment or how this varies between heterosexual and non-heterosexual men [4].

Rosser et al. in the Restore-1 study found that sexual history is noted in only 8.8% of patients [52]. Knowing a patient’s sexual orientation may help clinicians undertake more individualized discussions on risk vs benefits associated with each treatment modality, so that patients can make an informed decision on cancer-directed treatment. It has been suggested that radiotherapy for GBM engaging in insertive anal intercourse and surgery for GBM engaging in receptive anal intercourse might be reasonable treatment approaches. These recommendations are based on the assumption that surgery has higher rates of erectile dysfunction than radiotherapy, and radiotherapy results in higher rectal adverse effects than surgery [4]. The side effect profiles of these therapies are corroborated by the Prostate Cancer Outcomes Study (PCOS), which included 3533 men. The study revealed that prostatectomy was associated with a higher rate of erectile dysfunction at 2 years (OR 3.46, 95% CI 1.93–6.17) and 5 years (OR 1.96, 95% CI 1.05–3.63) after surgery in comparison to radiotherapy. However, there were no long-term differences at 15 years after therapy between the two groups [53].

The Restore-1 trial evaluated the experiences of discrimination in treatment faced by prostate cancer patients who identify as sexual and gender minorities. The study involved 192 participants, gay, bisexual, or transgender, in the United States. The participants were recruited from North America’s largest online cancer support group and were asked to complete an online survey. Discrimination in treatment was measured using the Everyday Discrimination Scale (EDS), which had been adapted for use in medical settings [54,55]. Almost half of the participants (46%) reported experiencing at least one discriminatory behavior, including being talked down to (25%), receiving poorer care than other patients (20%), being treated as inferior (19%), and having providers appear afraid of them (10%). Most participants rated the discrimination as rare or occasional, but 20% reported it as more common. The discrimination was mostly attributed to the participants’ sexual orientation, and to providers being arrogant or too pressed for time [56]. It remains unclear what effects on prostate cancer treatment these forms of discrimination have on sexual minorities.

Prostate cancer treatment in transgender women is generally similar to treatment received by cis-gender men [5]. Radical prostatectomy after GAS is not contraindicated [41]. As some transgender women may have undergone penectomy coupled with neovagina creation, there are resulting changes in anatomic landmarks [57]. Therefore, a treating surgeon must be aware of the altered anatomy between the prostate, neovagina, and rectum to perform a successful radical prostatectomy [58]. Moreover, the prostate is usually atrophied if a patient has undergone previous GAHT. In a small study with 14 patients on estrogen therapy, average prostate volume was 14.19 cm [59], which makes the landmarks for surgery less clear and radical prostatectomy more technically challenging. A smaller prostate requires tailored care with radiation therapy as well. Excessive radiation should be avoided as it can lead to neovaginal stenosis [41]. In cases of brachytherapy, seed implantation in the small prostate gland can increase the risk of toxicity to adjacent organs [60]. In TW who already are surgically castrated or have castrate levels of testosterone secondary to GAHT, androgen deprivation therapy (ADT) will not provide significant benefits. Second generation androgen receptor targeted therapies, such as enzalutamide or abiraterone, might be better options in this patient population [41]. However, there is no clear date to prove their benefit.

## 6. Quality of Life following Prostate Cancer Treatment in Sexual Minorities

There has been increasing recognition of sexual minority groups and the disparities faced by members of this population. Consequently, more qualitative and quantitative studies (Table 1) have been conducted to gain a better understanding of the effect of prostate cancer treatment on quality of life of patients from sexual and gender minority groups [7]. Although healthcare systems are shifting away from a heteronormative approach towards a more comprehensive and affirming method, recent research indicates that only a small percentage of oncology practitioners, including physicians, nurse practitioners, and nurses, feel adequately informed to address healthcare disparities faced by sexual and gender minorities [61].

Although progress in refining and creating new questionnaires to assess quality-of-life measures in GBM has been made, most studies use instruments, such as the Expanded Prostate Cancer Index (EPIC), 12-Item Short Form Health Survey (SF-12) and FACT-P, to assess the quality-of-life outcomes of prostate cancer. These questionnaires measure the effects of treatment on sexual, urinary, bowel and hormonal function, and perceived bother. They can be indicative of overall health-related quality of life in patients dealing with prostate cancer [64]. These questionnaires, however, are focused on heterosexual men and do not adequately capture quality of life outcomes in GBM and TW [51,65].

A systemic literature review of 88 qualitative, quantitative, and case studies focused on prostate cancer in GBM and TW revealed that GBM appear to have worse urinary and bowel function, but better sexual outcomes than published norms [24]. These results were confirmed in the Restore-2 randomized controlled clinical trial, the largest study with patients from sexual minority groups including 401 gay and bisexual prostate cancer patients [63]. As compared with heterosexual men on the EPIC-50, Restore-2 participants had worse urinary, bowel, and hormonal function, but better sexual function (*p* < 0.05). In addition, on the FACT-P, sexual minority patients scored significantly worse on physical, social, emotional, prostate-specific and overall wellbeing [63]. Thus, multiple methods of measurement have revealed disparities in quality-of-life outcomes in GBM.

Studies suggest that better sexual function in GBM may be due to differences in their sexual behavior in being more open, innovative, and committed to use strategies to accommodate the sexual effects of treatment (e.g., changes in sex roles). In addition, a greater percentage of heterosexual patients than sexual minority patients may not be sexually active which may also influence the outcomes [62]. The worse scores on mental health may be associated with minority stress theory [66], less familial and social support [67,68], and poorer experience in treatment [69].

Health disparities have important implications for clinical practice. While prostate cancer affects GBM in many of the same ways as heterosexual men, GBM prostate cancer survivors face unique challenges. When discussing treatment options, clinicians need to review the differential effects of treatment on insertive and receptive sexual functioning [70]. They include loss of the prostate as a site for sexual pleasure in receptive sex [28,67], loss of ejaculate (which is more central in gay sex) [71], persistent rectal irritation or pain sufficient to prevent receptive anal sex [21,72], and erections too weak for insertive anal sex [73].

There have been several recent advancements made in the study of quality-of-life outcomes of GBM. In early 2022, two new questionnaires were developed to enhance the assessment of sexual function outcomes in GBM after prostate cancer treatment. One of these questionnaires, known as the Sexual Minorities and Prostate Cancer Scale (SMACS), contains 37 items and was developed and validated as part of the Restore-2 study at the University of Minnesota [74]. It enables the assessment of sexual satisfaction, confidence, frequency of sexual issues, urinary incontinence during intercourse and problematic receptive anal intercourse. The other questionnaire was developed at the University of British Columbia and contains 13 questions that include inclusive inquiries regarding insertive and receptive anal intercourse, as well as masturbation practices. This scale has been pilot-tested in healthy GBM and those receiving prostate cancer treatment, but has not yet been validated [75]. These two questionnaires offer potential new tools for effectively evaluating sexual quality of life in GBM following prostate cancer treatment [74,75].

The data on quality-of-life outcomes for transgender women treated for prostate cancer are mostly limited to case studies, and as such, there is a paucity of population-based or clinical trial data [5]. Despite being eligible for enrollment in the aforementioned Restore-2 study, no transgender women participated [63]. With an increasing number of people identifying as transgender or gender non-conforming, it is crucial for healthcare professionals to conduct studies that evaluate the health care and quality-of-life outcomes of this growing population.

## 7. Clinical Implications and Future Directions

In this review, we presented a comprehensive overview of prostate cancer in sexual minorities to aid healthcare professionals, such as primary care providers, urologists, medical oncologists, palliative care physicians, psychiatrists, nurses, and support groups, in understanding the unique challenges faced by this marginalized patient population diagnosed with prostate cancer. By delving into the specifics of prostate cancer screening, diagnosis, treatment, and quality of life, healthcare professionals can offer more culturally appropriate care to their patients. Nonetheless, further research is necessary to explore how prostate cancer impacts sexual minority groups in various ethnicities, cultures, and regions worldwide, as most of the data presented in this review originates from studies conducted in European and North American countries. We have highlighted the nuances of prostate cancer treatment and its impact on the quality of life of sexual minority patients. Nevertheless, additional studies are needed to guide the nursing and supportive care of sexual minorities following prostate cancer treatment.

## 8. Conclusions

Growing evidence suggests disparities in diagnosing and treating prostate cancer in sexual minorities with resulting disparities in quality-of-life outcomes following treatment. However, despite several recent studies, there continues to remain a paucity of data in this growing population. Studies on best practices are required to transform clinical care to be more culturally responsive to the needs of sexual minority patients with prostate cancer.

## Figures and Tables

**Table 1:** Studies conducted on the quality-of-life outcomes in sexual minority patients following prostate cancer treatment.

Study	Number of Patients/Type of Contribution	Outcome	Ref
Restore-1 Survey	175 Gay/Homosexual (90.7%)	-Worse mental health -Better physical health -Challenging sexual recovery data	[62]
	18 Bisexual/Others (9.3%)		
Restore-2 Study	371 Gay/Homosexual (92.5%)	-Worse urinary, bowel and hormonal function -Better sexual function	[63]
	30 Bisexual (7.5%)		
Review of Literature on GBM and TW	23 Case Reports in GBM	-Poor urinary, bowel function -Poor overall quality-of-life -Better sexual function	[21]
	5 Case Reports in TW		
	9 Reviews		
	11 Qualitative Study report		
	19 Quantitative Study report		
	5 Commentary		
	13 Clinical Observation		
	2 others		
Review of Literature on TW	10 case reports	-Lack of information on the quality-of-life outcome in TG patients	[4]
	6 cohort studies		
	4 specialist opinion		
	4 systemic reviews		
